# Multicellular Tumor Spheroids for Evaluation of Cytotoxicity and Tumor Growth Inhibitory Effects of Nanomedicines *In Vitro*: A Comparison of Docetaxel-Loaded Block Copolymer Micelles and Taxotere®

**DOI:** 10.1371/journal.pone.0062630

**Published:** 2013-04-23

**Authors:** Andrew S. Mikhail, Sina Eetezadi, Christine Allen

**Affiliations:** 1 Leslie Dan Faculty of Pharmacy, University of Toronto, Toronto, Ontario, Canada; 2 Institute of Biomaterials and Biomedical Engineering, University of Toronto, Toronto, Ontario, Canada; 3 Spatio-Temporal Targeting and Amplification Radiation Response (STTARR) Innovation Centre, Toronto, Ontario, Canada; The Ohio State University, United States of America

## Abstract

While 3-D tissue models have received increasing attention over the past several decades in the development of traditional anti-cancer therapies, their potential application for the evaluation of advanced drug delivery systems such as nanomedicines has been largely overlooked. In particular, new insight into drug resistance associated with the 3-D tumor microenvironment has called into question the validity of 2-D models for prediction of *in vivo* anti-tumor activity. In this work, a series of complementary assays was established for evaluating the *in vitro* efficacy of docetaxel (DTX) -loaded block copolymer micelles (BCM+DTX) and Taxotere® in 3-D multicellular tumor spheroid (MCTS) cultures. Spheroids were found to be significantly more resistant to treatment than monolayer cultures in a cell line dependent manner. Limitations in treatment efficacy were attributed to mechanisms of resistance associated with properties of the spheroid microenvironment. DTX-loaded micelles demonstrated greater therapeutic effect in both monolayer and spheroid cultures in comparison to Taxotere®. Overall, this work demonstrates the use of spheroids as a viable platform for the evaluation of nanomedicines in conditions which more closely reflect the *in vivo* tumor microenvironment relative to traditional monolayer cultures. By adaptation of traditional cell-based assays, spheroids have the potential to serve as intermediaries between traditional *in vitro* and *in vivo* models for high-throughput assessment of therapeutic candidates.

## Introduction

It has become increasingly clear that resistance to chemotherapy is not only facilitated by processes at the cellular level, but also by mechanisms associated with the tumor microenvironment [1], [2]. In growing tumors, the heterogeneous architecture of the vasculature, irregular blood flow, large intervascular distances and nature of the extracellular matrix limit the access of cells to oxygen, nutrients, and systemically administered therapies [3], [4]. Within the tumor interstitium, gradients in the rate of cell proliferation are established wherein rapidly dividing cells reside close to the tumor vasculature and quiescent cells are situated deep within the extravascular space. However, many anti-neoplastic agents exert limited toxicity against slowly- or non-proliferating cells and are less effective in the hypoxic and acidic microenvironments of poorly perfused tissues [5], [6]. These therapeutic limitations are exacerbated by high interstitial fluid pressure which inhibits the penetration of chemotherapeutic agents through the tumor interstitium by limiting convective transport [7]. As a result cells located distant from blood vessels may be less sensitive to treatment and also be exposed to sub-therapeutic drug concentrations.

The use of *in vitro* cell culture is critical in drug discovery and formulation development for rapid identification of lead candidates and for investigating mechanisms of drug efficacy at the cellular and molecular levels. In contrast to *in vivo* tumor models, *in vitro* cultures are better suited for systematic studies of formulation parameters in a highly controlled environment. However, cytotoxic effects observed in conventional monolayer cultures often fail to translate into similar effects *in vivo* [8], [9]. This is due to the inherent inability of 2-D cultures to account for mechanisms of drug resistance and transport restrictions associated with the 3-D tumor microenvironment. As such, there is increasing interest in applying 3-D *in vitro* models that enable rapid, high throughput screening of drug formulations for selection of lead candidates to move forward to *in vivo* evaluation [10]–[12].

**Figure 1:**
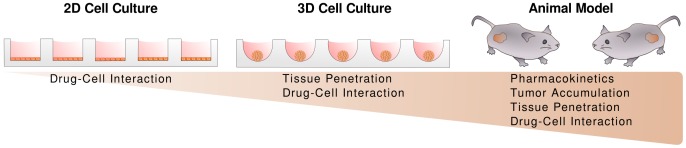
3-D cultures as intermediary between 2-D cultures and animal models. Intermediate in complexity, 3-D cultures permit the systematic, high-throughput assessment of formulation properties in a controlled environment that approximates important properties of *in vivo* tumors in the absence of complex parameters which may confound data interpretation.

As depicted in Figure 1, 3-D tissue cultures such as MCTS serve as an intermediary between the oversimplified structure of monolayer cultures and the highly complex nature of *in vivo* tumors. Spheroid cultures possess a complex network of cell-cell contacts and advanced extracellular matrix development, as well as pH, oxygen, metabolic and proliferative gradients analogous to the conditions in poorly vascularized and avascular regions of solid tumors [13]–[15]. In general, a spheroid is comprised of an outer region of proliferating cells which surrounds intermediate layers of quiescent cells and, if the spheroid is large enough, a necrotic core. This arrangement parallels the radial organization of tissues surrounding tumor blood vessels. To date, a variety of 3-D *in vitro* tissue models have been applied for the study of anticancer therapies including natural and synthetic tissue scaffolds [16], [17], multicellular layers [18]–[22], and multicellular tumor spheroids [16], [23], [24]. MCTS are particularly relevant in the development of nanomedicines since the penetration of the encapsulated drug in tumor tissues may be significantly altered by properties of the delivery vehicle. To date, however, there remain limited examples of the use of MCTS for the evaluation of nanomedicines [25]–[29].

**Figure 3:**
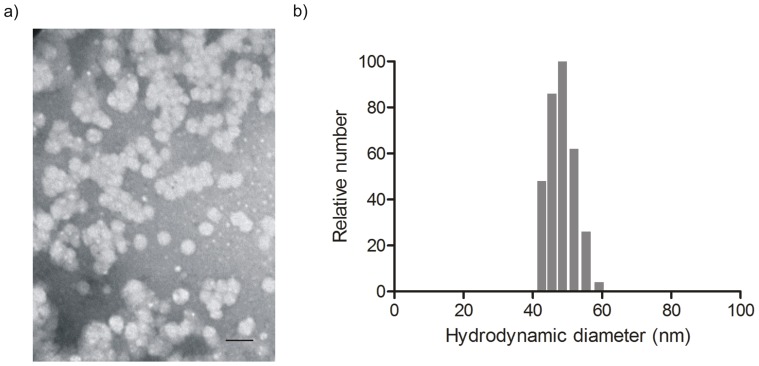
Characterization of micelle morphology and size. a) Transmission electron micrograph (Scale bar in represents 100 nm) and b) size distribution of BCM+DTX as determined by dynamic light scattering at 37°C.

**Figure 2:**
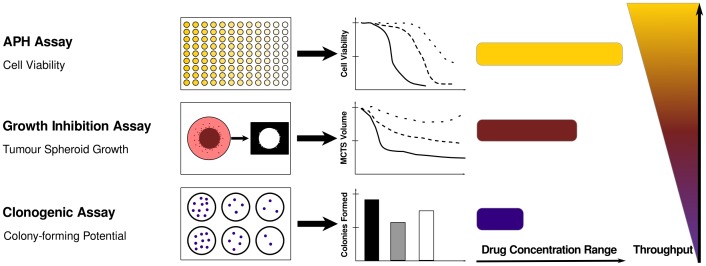
*In vitro* assays used in this study for analysis of formulation efficacy in spheroids.

DTX is a potent chemotherapeutic agent that is administered as Taxotere® (Sanofi-Aventis) and used for treatment of cancers of the breast, prostate, lung, head and neck, and stomach [30]. DTX is also being investigated in a phase II clinical trial for treatment of metastatic colorectal adenocarcinoma in combination with gemcitabine and has been investigated as a single agent for treatment of cervical cancer [31], [32]. However, Taxotere® is known to be associated with significant side effects that can require reduction of the administered dose [33]. Encapsulation of chemotherapeutic agents within biocompatible nanosystems such as block copolymer micelles (BCMs) has proven to be a promising approach for mitigating the burden of toxicity on normal tissues and increasing tumor-specific drug accumulation [34]. The primary objective of this study was to adapt and apply traditional cell-based assays in a systematic and complementary manner for the evaluation of Taxotere® and a DTX-containing nanomedicine in both monolayer and MCTS cultures (Figure 2).

## Materials and Methods

### Materials

Methoxy poly(ethylene glycol) (CH_3_O-PEG-OH; Mn = 5000, Mw/Mn = 1.06) was obtained from Sigma-Aldrich (Oakville, ON, Canada). ε-Caprolactone and dichloromethane (Sigma-Aldrich) were dried using calcium hydride prior to use. Hydrogen chloride (HCl) (1.0 M in diethyl ether), N,N-dimethylformamide (DMF), diethyl ether, hexane and acetonitrile (Sigma-Aldrich) were used without further purification. Alexa Fluor 488 (AF488) carboxylic acid succinimidyl ester was purchased from Molecular Probes (Eugene, OR). The hypoxia marker, EF5, and Cy5-conjugated anti-EF5 antibody were purchased from the Department of Radiation Oncology, University of Pennsylvania, (Philadelphia, PA). DTX was purchased from Jari Pharmaceutical Co. (Jiangsu, China).

### Synthesis of CH_3_O-PEG-*b*-PCL (PEG-*b*-PCL) Copolymers

PEG-*b*-PCL copolymer was prepared as previously described [35]. Briefly, CH_3_O-PEG-OH was used to initiate the ring-opening polymerization of *ε*-CL in the presence of HCl. The reaction was carried out for 24 h at room temperature prior to termination by addition of triethylamine (TEA) and precipitation in diethyl ether and hexane (50∶50, v/v%). The product was dried under vacuum at room temperature.

### Preparation and Characterization of BCM+DTX

PEG-*b*-PCL copolymers and DTX were dissolved at a copolymer:drug weight ratio of 20∶1 in DMF and stirred for 30 min. DMF was evaporated under N_2_ at 30°C and residual solvent was removed under vacuum. Dry copolymer-drug films were then heated to 60°C in a water bath prior to the addition of PBS buffer (pH 7.4) at the same temperature. Resultant micelle solutions were vortexed, stirred for 24 h at room temperature and finally sonicated (Laboratory Supplies Co., NY) for 1 h. Undissolved drug crystals were removed by centrifugation at 4400 g for 12 min (Eppendorf 5804R). The final copolymer concentration was 10 mg/mL. The amount of physically entrapped DTX in BCM samples was determined by HPLC analysis (Agilent series 1200) with UV detection (Waters 2487) at a wavelength of 227 nm. An XTerra C18 reverse phase column was employed with ACN/water (60/40, v/v%) as the mobile phase. Drug loading was quantified using a calibration curve generated from a series of DTX standards.

### Sizing of BCM+DTX

The average hydrodynamic diameter of the BCMs was determined by dynamic light scattering (DLS) using a 90Plus Particle Size Analyzer (Brookhaven Instruments Corp., Holtsville, NY) at an angle of 90° and temperature of 37°C. The samples were diluted to a copolymer concentration of 0.5 mg/mL prior to measurement. Analysis was performed using the 90Plus Particle Sizing Software.

### Transmission Electron Microscopy (TEM)

BCMs were observed by TEM using a Hitachi 7000 microscope operating at an acceleration voltage of 75 kV (Schaumburg, IL). Samples were diluted in double distilled water immediately prior to analysis and negatively stained with a 1% uranyl acetate (UA) solution. The final copolymer concentration was 0.5 mg/mL. The samples were then deposited on copper grids that had been pre-coated with carbon and negatively charged (Ted Pella Inc., Redding, CA) and briefly air-dried prior to analysis.

### Drug Release

The release of DTX from BCMs and Taxotere® was analyzed using a dialysis method. Aliquots (1 mL) of BCM+DTX, DTX in DMSO, and Taxotere® were placed in individual dialysis bags (MWCO 2 kDa, Spectra/Por, Rancho Dominguez, CA) and dialyzed separately against 100 mL of PBS at pH 7.4 in an incubator at 37°C ensuring that sink conditions were maintained. At selected timepoints, 50 µL samples were withdrawn from the dialysis bags and DTX content was measured by HPLC as described above.

### Tissue Culture and Growth of MCTS

Human cervical (HeLa) and colon (HT29) (ATCC, Manassas, VA) cancer cells were incubated at 37°C and 5% CO_2_ in DMEM containing 1% penicillin-streptomycin and supplemented with 10% FBS. For growth of MCTS, cells were suspended using trypsin-EDTA and 2000 and 5000 HT29 and HeLa cells were seeded onto non-adherent 96-well round-bottomed Sumilon PrimeSurface™ plates (Sumitomo Bakelite, Tokyo, Japan), respectively, in 200 µL of media per well. During growth, 50% of the media was exchanged every other day. MCTS were grown for 7 days until they reached ∼ 500 µm in diameter before use.

### Immunohistochemical Analysis of MCTS

MCTS were washed in PBS and transferred onto a vinyl specimen mold (Cryomold®, Tissue-Tek, Sakura Finetek, CA) prior to addition of Tissue-Tek® O.C.T. compound (Sakura Finetek, Torrance, CA). MCTS were then submersed in an isopentane bath cooled by liquid nitrogen, cut into 5 µm thick sections using a microtome and mounted on glass slides. Histological staining was conducted for the identification of cellular proliferation (Ki67) and stained with hematoxylin and eosin (H&E). For identification of hypoxic regions, MCTS were incubated with 0.5 mM EF5 and soaked in PBS prior to cryosectioning. EF5 in the MCTS sections was identified by binding with cyanine-5-conjugated mouse anti-EF5 (1/50) antibody. The positive signal distribution for Ki67 was analyzed using a customized MATLAB® algorithm, as described previously [36]. Briefly, images containing Ki67-stained MCTS sections were thresholded for positive color intensity. Using a distance map, signal intensities were summed within three concentric regions of equidistant thickness (periphery, intermediate and core), each equivalent to 1/3 of the MCTS radius. The distribution of Ki67 positive signal is expressed as a percentage of total positive signal in the MCTS section.

### Measurement of MCTS Growth

Spheroids were imaged using a light microscope with a 10× objective lens (VWR VistaVision TM) connected to a digital camera (VWR DV-2B). The diameter and volume of MCTS were determined by measuring their cross-sectional area using an automated image analysis macro developed for use with the ImageJ software package (NIH, Bethesda, MD, Version 1.44 m). The automated method was validated by comparison to manual determination of spheroid diameter and volume (Figure S1). For the automated method, images were converted into 8-bit greyscale and the perimeter of an individual MCTS was recognized by an automated threshold function and the image converted to a 2-D mask. The area of the spheroid mask was recorded, applying an image of known scale as calibration. Finally, the volume of the MCTS was calculated by assuming a spherical shape as follows: V = 4/3*π*(d/2)^3^. Data was fit using the Gompertz equation for tumor growth as follows: V(t) = V(0)exp(α/β(1−exp(−β*t))) where V(t) is volume at time t, V(0) the initial volume and α and β are constants [37].

### Cytotoxicity in Monolayer and Spheroid Tissue Cultures

The cytotoxicity of BCM+DTX and Taxotere® in monolayer and spheroid cell cultures was determined using the established acid phosphatase (APH) assay which is based on quantification of cytosolic acid phosphatase activity [38]. For this assay, *p*-nitrophenyl phosphate is added in cell culture and hydrolyzed in viable cells to *p*-nitrophenol via intracellular acid phosphatase. Briefly, MCTS (one spheroid per well) and monolayer cultures (4000 cells per well) were treated with Taxotere® or BCM+DTX for 24 h over a range of drug concentrations. Following treatment, monolayers and spheroids were washed three times with fresh media and cultured for an additional 48 h. Monolayers and spheroids were then washed with PBS buffer prior to the addition of 100 µL of freshly prepared reaction buffer (2 mg/ml *p*-nitrophenyl phosphate (Sigma) and 0.1% v/v Triton-X-100 in 0.1 M sodium acetate buffer at pH 5.5). Following incubation for 2 h in the cell incubator, 10 µL 1 M sodium hydroxide was added to each well and cell viability was determined by measuring the UV absorbance at 405 nm using an automated 96-well plate reader (SpectraMax Plus 384, Molecular Devices, Sunnyvale, CA). Results were normalized to controls as follows: % viability = (A_treatment_ – A_media_)/(A_control_ – A_media_), where A = mean absorbance. All experiments were performed in triplicate.

### Growth Inhibition of MCTS

BCM+DTX or Taxotere® was administered to spheroids for 24 h at a DTX equivalent concentration of 2, 20 or 200 ng/mL. The culture media was replaced following the incubation period. Subsequently, half of the culture media was replaced by pipette every other day. Images of spheroids were captured using a light microscope with a 10× objective lens (VWR VistaVision™) connected to a digital camera (VWR DV-2B). Spheroid size was determined by measuring their 2-D cross-sectional area using the automated image analysis method described previously. The data are reported as the mean volume of six spheroids ± SD.

### Clonogenic Survival Assay

The clonogenic assay was used to determine the ability of single cells to replicate and form colonies (>50 cells) following exposure to BCM+DTX and Taxotere®. Single cell suspensions derived from monolayer and disaggregated spheroids were diluted in culture media and cells were plated in 6-well plates in desired numbers. MCTS were disaggregated by incubation in trypsin-EDTA for 10 min, followed by gentle agitation. Drug formulations were added immediately at a DTX equivalent concentration of 20 ng/mL. After treatment for 24 h, cells were washed with PBS and 2 mL of fresh media was added to each well. For treatment of intact spheroids, drug formulations were added directly into wells containing individual MCTS. After 24 h, MCTS were collected and rinsed in PBS, suspended as single-cell suspensions in fresh media following trypsinization, and seeded onto 6-well plates. Cells were incubated for 14–16 days prior to fixation with methanol and staining with 1% crystal violet solution. Colonies consisting of at least 50 cells were counted. The surviving fraction (SF) was expressed as the number of colonies divided by the product of the number of cells plated and the plating efficiency. The plating efficiency was determined by dividing the number of colonies formed by the number of cells plated for untreated controls.

## Results

### Characterization of BCM+DTX

**Figure 4:**
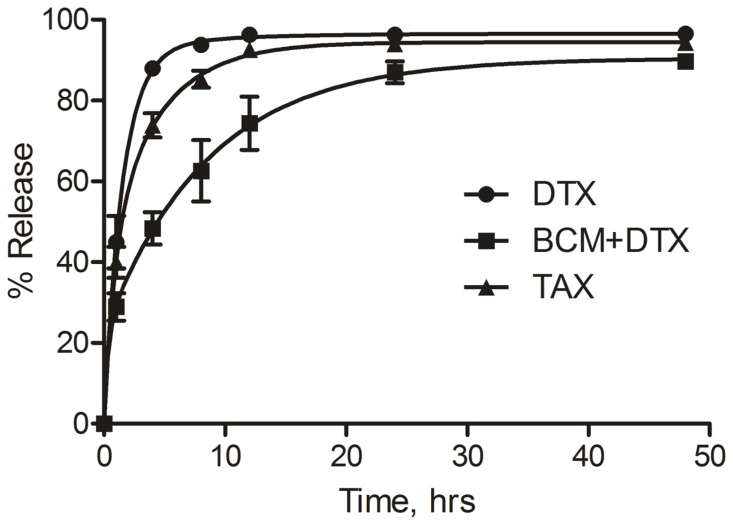
Drug release. **Release of docetaxel from dialysis bags containing BCM+DTX, Taxotere®, and DTX in DMSO, n = 3.**

PEG-*b*-PCL copolymer micelles containing physically encapsulated DTX were formulated with a spherical morphology (Figure 3a). The size distribution of the micelles was monomodal with an average hydrodynamic diameter of 49.2±2.3 nm (Figure 3b). Drug loading resulted in a final DTX equivalent concentration of 258.7±35.5 µg/mL at a loading efficiency of 52.7±7.1%. Release of DTX from BCMs occurred over the course of 24 h wherein 74% of the drug was released by 12 h. In contrast, the release of docetaxel from Taxotere® was complete by 12 h (Figure 4).

### Growth of MCTS

**Figure 5:**
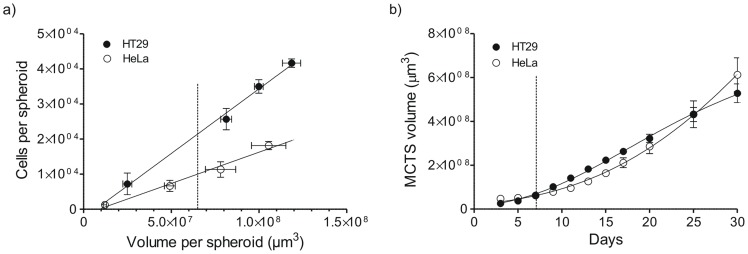
Spheroid packing density and growth. a) Cells per HeLa and HT29 spheroid of given volume, n = 12. b) Growth of HeLa and HT29 spheroids, n = 6. Data was fit using the Gompertz equation for tumor growth. The dashed lines indicate spheroid properties used in the studies.

Spheroids were grown using a modified liquid overlay technique by seeding HT29 or HeLa cells onto non-adherent U-bottom tissue culture wells without the use of an agarose surface coating. MCTS were spherical, followed a sigmoidal growth profile, and were grown until a diameter of ∼500 µm was reached prior to use (Figure 5).

### Cytotoxicity in Monolayer and MCTS Culture

**Figure 6:**
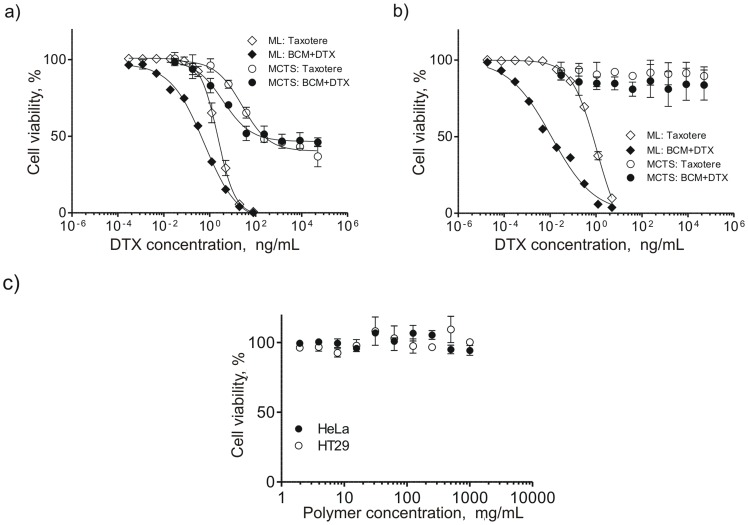
Cytotoxicity of Taxotere® and BCM+DTX in spheroid and monolayer cultures. Viability of a) HeLa and b) HT29 cells cultured as monolayers and spheroids as measured using the APH assay. Data is expressed as the percent viability relative to untreated controls and fit to the Hill equation. c) Cytotoxicity of blank PEG-*b*-PCL micelles as a function of copolymer concentration. Each plot represents the mean of three independent experiments ± SD (n = 3).

Cell viability following exposure to BCM+DTX or Taxotere® was assessed using the APH assay (Figure 6). This assay was validated by assessment of the relationship between UV absorbance and cell number in both monolayer and spheroid cultures. As shown in Figure S2, a linear relationship was obtained. A well-established tetrazolium salt-based assay (WST-8) was also evaluated and did not yield a similar correlation (Figure S3). Spheroid cultures were substantially less sensitive to BCM+DTX and Taxotere® relative to their monolayer counterparts. HeLa cells were less responsive to treatment with either BCM+DTX or Taxotere® than HT29 cells in monolayer culture. However, in spheroid culture, HT29 cells were less sensitive to treatment. The IC_50_ of HeLa and HT29 monolayer cultures treated with BCM+DTX were 0.37+/−0.01 and 0.01+/−0.004 ng/mL, respectively. When treated with Taxotere®, the IC_50_ of HeLa and HT29 monolayer cultures were 2.2+/−0.5 and 0.09+/−0.01 ng/mL, respectively. The IC_50_ of HeLa cells cultured as MCTS was 1396±198 ng/mL for BCM+DTX and 1558±103 ng/mL for Taxotere® whereas HT29 MCTS maintained a viability above 80% at all drug concentrations.

### Inhibition of MCTS Growth

**Figure 7:**
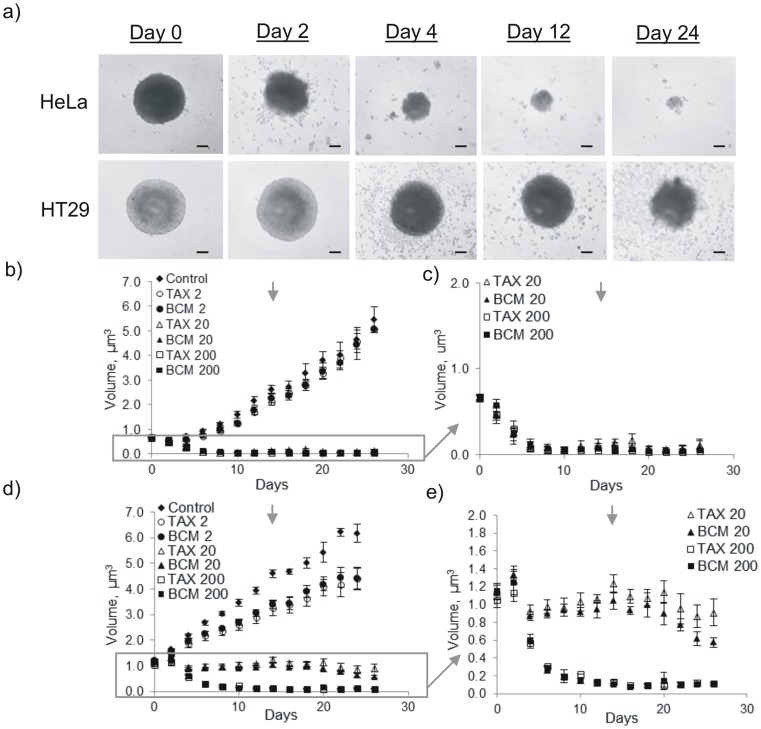
Inhibition of spheroid growth. a) Sequential images of the same HeLa and HT29 spheroids following treatment with BCM+DTX at a concentration of 20 ng/mL. Bars represent 100 µm. Growth inhibition of HeLa (b,c) and HT29 (d,e) MCTS by BCM+DTX and **Taxotere**® at concentrations of 2, 20 and 200 ng/mL. Cells were re-treated after two weeks (arrow). Box represents expanded region of plots b) and d). Data is expressed as the mean volume of six spheroids (n = 6) ± SD. “*” represents a significant difference between BCM 20 and TAX 20, p<0.05.

MCTS volume was plotted over a 30 day period following a 24 h incubation with 2, 20, and 200 ng/mL BCM+DTX or Taxotere® (Figure 7). The growth of HeLa MCTS was completely impeded following incubation with DTX concentrations of 20 and 200 ng/mL. No significant difference in growth was observed following exposure to 2 ng/mL of DTX relative to untreated controls. In the case of HT29 MCTS, incubation with 20 ng/mL of BCM+DTX and Taxotere® only resulted in a partial reduction in MCTS volume. Similarly to HeLa MCTS, complete inhibition of growth was observed following incubation with 200 ng/mL of drug. Unlike HeLa MCTS, however, a slight growth delay was also observed at 2 ng/mL. Interestingly, following re-treatment on day 14 at a DTX concentration of 20 ng/mL, BCM+DTX demonstrated greater inhibition of spheroid growth in HT29 cultures than Taxotere®.

### Immunohistochemistry

**Figure 8:**
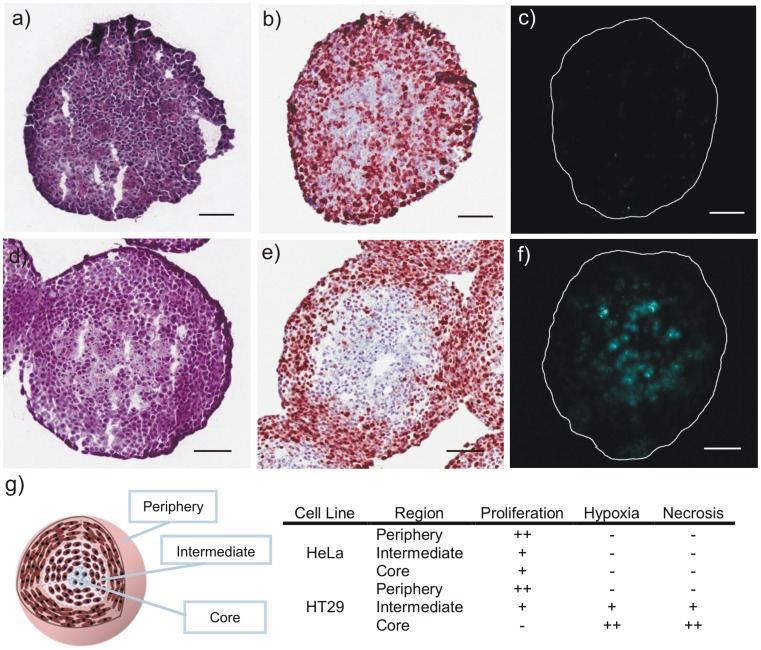
Histological assessment of spheroid microenvironment. HeLa (a–c) and HT29 (d–f) MCTS cross-sections stained with H&E (a, d), Ki67 proliferation marker (b, e) and EF5 (c, f), a marker of hypoxia. Scale bars represent 100 µm. g) Properties of the spheroid microenvironment and their spatial distribution. “++”, “+”, and “–”, indicate high, intermediate and low levels of the corresponding feature, respectively.

**Figure 9:**
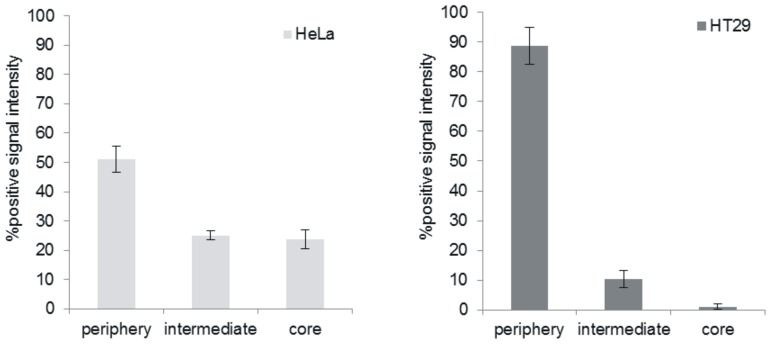
Spatial distribution of proliferating cells in spheroids. Ki67 positive signal distribution relative to radial position in a) HeLa and b) HT29 MCTS as a percent of total positive stain, n = 6.

**Figure 10:**
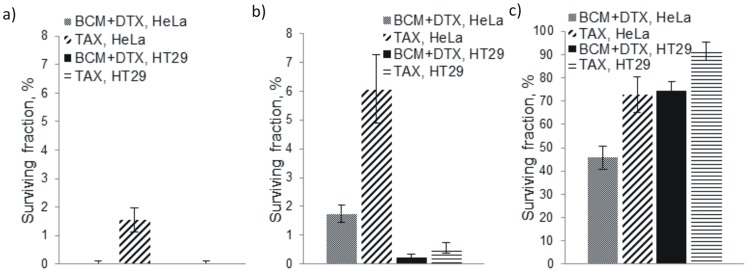
Clonogenic potential of cells following treatment. Clonogenic survival of HeLa and HT29 cells following 24 h treatment with 20 ng/mL of BCM+DTX or Taxotere® as a) monolayers, b) disaggregated spheroids and c) intact spheroids.

Immunohistochemical analysis of MCTS cross-sections was performed in order to identify regions of necrosis, cellular proliferation and hypoxia (Figure 8). Staining with the proliferation marker Ki67 revealed a greater proportion of proliferative cells in HeLa MCTS relative to HT29. Quantitative image analysis revealed that 88.6% of proliferating cells were located within the periphery of HT29 MCTS (Figure 9). In contrast, only 51% of the total proliferating cells were located in the periphery of HeLa MCTS and 25% and 24% were located in the intermediate region and core, respectively. Signs of necrosis were visible following staining with H&E in HT29 MCTS. Incubation of MCTS with EF5 allowed for identification of regions of hypoxia following exposure to Cy5-conjugated anti-EF5 antibody. Hypoxic conditions were observed primarily in the core and intermediate regions of HT29 MCTS. In contrast, HeLa MCTS did not demonstrate any regional hypoxia. The relative distributions of cellular proliferation, hypoxia and necrosis in the MCTS are summarized in Figure 10.

### Clonogenic Survival

The surviving fractions (SF) of HeLa and HT29 cells were determined following treatment with BCM+DTX or Taxotere® as monolayer and MCTS cultures (Figure 10).

The SF was higher for all intact MCTS cultures relative to monolayers. HeLa cells were less sensitive to treatment than HT29 when cultured as monolayers, but more sensitive than HT29 cells when the cells were exposed to treatment as MCTS. In all cases, the SF was lower when treated with BCM+DTX compared to Taxotere®. Furthermore, cells exposed to treatment immediately following MCTS disaggregation demonstrated residual resistance to both BCM+DTX and Taxotere®.

## Discussion

In recent years, the tumor microenvironment has been implicated in the coordination of tumor growth, metastasis and resistance to anti-cancer therapies [39], [40]. As such, effective evaluation of novel therapeutic agents requires the use of tissue models which closely mimic native conditions within the intratumoral space. Yet, the vast majority of chemotherapeutic agents are screened for cytotoxic effects in monolayer cultures which do not account for critical mechanisms of drug resistance associated with the tumor microenvironment. Consequently, these models poorly predict a drug’s therapeutic efficacy *in vivo* [8]. In contrast, 3-D MCTS better approximate the state of cancer cells in their native environment and thus can be used to more accurately estimate a drug’s therapeutic potential. A variety of methods have been used to grow MCTS for use in cancer research including spinning culture flasks [41], hanging drops [42], liquid overlay on agarose [43], micropatterned plates [44], and recently, using inter-cellular linkers [45]. However, many of these techniques are impractical, time-consuming, and involve delicate handling procedures, limiting the use of the MCTS model in drug screening and development. In addition, practical application of traditional cell-based assays in MCTS cultures remains poorly established. In the current study, the performance of BCM+DTX and Taxotere® was evaluated by adaptation of conventional cytotoxicity and survival assays in monolayer and MCTS cultures using a robust MCTS culture technique.

MCTS grew according to sigmoidal growth patterns reflective of tumor growth *in vivo* (Figure 5) and possessed histological features similar to those of the native tumor microenvironment including gradients in cell proliferation and regions of hypoxia and necrosis (Figure 8, Figure S4). Cells grown in spheroid cultures demonstrated considerably greater resistance to treatment with BCM+DTX or Taxotere® relative to cells grown in monolayer cultures. This may be a result of the limited exposure of cells within MCTS to treatment due to poor penetration of DTX or BCMs, the limited sensitivity of cells within MCTS to DTX due to a reduction in cellular proliferation and/or resistance associated with 3-D cell adhesion (i.e. contact effect). In a study by Kyle et al., the penetration half-depth (the depth from the surface at which the amount of drug falls to half of its maximum concentration) of DTX in multicellular layers was found to be <25 µm following a 2 h incubation at a concentration of 0.3 µM [46]. Peak tissue levels did not increase proportionally following a 10-fold increase in drug concentration although the depth of penetration was improved indicating partial saturation of tissue binding. Therefore, it is likely that high intracellular binding and consumption of DTX by peripheral cells in the MCTS limits the toxicity to cells distant from the surface. For drugs which are rapidly consumed by cells, encapsulation in BCMs which minimize interactions and uptake by cells may improve drug penetration [47]. For example, Pun et al. reported ameliorated penetration of doxorubicin into MCTS when encapsulated in triblock copolymer micelles [25]. However, BCMs which penetrate poorly through tissues may limit the penetration of the encapsulated drug. Overall, the extent to which the BCMs influence drug penetration will depend on the relative rates of drug release and BCM penetration in the MCTS. We have previously found that PEG-*b*-PCL BCMs of 55 nm diameter can achieve a homogeneous distribution in MCTS following a 24 h incubation (unpublished data).

In addition to potential limitations in MCTS penetration associated with the drug and BCMs, the discrepancy between MCTS and monolayer cytotoxicity may also be a result of drug resistance imparted by the MCTS microenvironment. A marked decrease in the proportion of proliferating cells was observed in MCTS with increasing depth from the surface (Figure 9). Since DTX exerts its therapeutic effect on cycling cells, cells located near the MCTS surface will respond to treatment similarly to cells cultured as monolayers. By contrast, quiescent cells that are located in the intermediate and core regions of the MCTS will be less sensitive to treatment. This notion is supported by the observation that cells exposed to treatment immediately following disaggregation of MCTS demonstrated greater clonogenic survival than monolayer cells, but less than cells treated as intact MCTS. Therefore, there exists a population of cells within the MCTS that is more resistant to treatment than cells cultured as monolayers even in the absence of any physical barrier to drug penetration. As such, the limited sensitivity of MCTS to treatment is likely a result of both restricted transport and mechanisms of drug resistance associated with the MCTS microenvironment.

The extent to which culturing cells as MCTS influenced the therapeutic effect of BCM+DTX and Taxotere® relative to monolayers was found to be cell-line specific. In monolayer cultures, BCM+DTX and Taxotere® demonstrated greater cytotoxicity against HT29 cells relative to HeLa cells. In contrast, culturing cells as MCTS imparted a greater enhancement in therapeutic resistance (i.e. greater increase in IC_50_) to HT29 cells than to HeLa cells. We have previously shown significantly greater penetration of BCMs into HeLa MCTS than HT29 MCTS due to the former’s lower cell packing density and large intercellular channels (unpublished data). In the current study, significant cell line-dependent differences in MCTS microenvironment were observed. Limited permeability of HT29 MCTS and/or high consumption of oxygen by peripheral cells was reflected by the presence of central hypoxia and necrosis. Importantly, HT29 MCTS contained a greater proportion of non-proliferating cells relative to HeLa MCTS. It is likely that some quiescent cells within the MCTS retained their clonogenic potential following exposure to sub-therapeutic amounts of DTX and were capable of recommencing proliferative activity when re-plated as monolayers. The greater clonogenic potential of HeLa cells following disaggregation of MCTS relative to HT29 cells likely reflects the greater sensitivity of HT29 monolayer cells to DTX rather than greater residual resistance of MCTS-derived HeLa cells.

One of the important advantages of the MCTS model is that it allows for treatment efficacy to be observed over an extended period of time. In order to evaluate the potential of surviving cells to repopulate MCTS, the growth of MCTS following treatment with BCM+DTX and Taxotere® was evaluated for 28 days with treatment re-applied after 14 days. The results of this study demonstrate both dose- and time-dependent changes in MCTS growth following incubation with the drug formulations. Near complete elimination of HeLa MCTS was observed following treatment at 20 ng/mL or greater with either BCM+DTX or Taxotere®. In contrast, only partial growth inhibition was observed in HT29 MCTS when exposed to the same concentration. This observation is consistent with the results obtained from the cytotoxicity and clonogenic assays in which HT29 MCTS demonstrated greater resistance to treatment relative to HeLa MCTS. A slight inhibitory effect in HT29 MCTS following administration of DTX formulations at 2 ng/mL was likely due to the cytotoxicity and shedding of surface cells, consistent with the response of HT29 cells to treatment in monolayer cultures. In addition, the apparent discrepancy between the limited cytotoxicity in HT29 spheroids revealed using the APH assay (measured 2 days post drug incubation) and the marked growth inhibition at 20 ng/mL is consistent with the observed 4 day delay in growth inhibitory effect. Interestingly, little difference in spheroid growth inhibition was observed between BCM+DTX and Taxotere® following initial treatment. It should be noted, however, that following retreatment after 14 days of culture, BCM+DTX demonstrated a greater growth inhibitory effect relative to Taxotere®.

Several factors may have contributed to the greater cytotoxicity of BCM+DTX relative to Taxotere® in monolayer and MCTS cultures. It has been hypothesized that DTX is taken up more rapidly by cells following release from BCMs in close proximity to the cell membrane due to an increase in the local transmembrane concentration gradient [48]–[50]. Slower efflux of BCM-encapsulated DTX relative to free DTX, by avoidance of membrane efflux pumps, may also contribute to the greater therapeutic effect of the DTX-loaded BCMs [51]–[53]. While these results are promising, further investigation is required to fully elucidate the mechanism of cytotoxicity that lead to enhanced therapeutic effects of BCM+DTX relative to Taxotere® *in vitro*.

Overall, as outlined in Figure 2, each of the three assays employed in this study is unique and together they provide complementary information on the therapeutic potential of drug formulations. Importantly, comparison of results obtained in monolayer and spheroid cultures demonstrated the important influence of the microenvironment and 3-D tissue structure on formulation efficacy. Therefore, 3-D cultures such as MCTS may serve as important tools for investigating the performance of nanomedicines in environments that more closely mimic intratumoral conditions *in vivo*. However, while spheroids share several important structural and microenvironmental properties with native tumors, there are important differences which may limit the extent to which this *in vitro* model can be used to predict drug efficacy *in vivo*. Notably, the MCTS model does not account for the potential influence of convective flow or presence of stromal cells on drug and nanoparticle transport. Despite these limitations, evaluation of formulation efficacy in spheroids rather than monolayer cultures is expected to more accurately reflect therapeutic performance *in vivo*.

## Supporting Information

Figure S1 **Measurement of spheroid volume.** a) Schematic representation of the analysis process using a macro developed for ImageJ (version 1.44 m). b) Correlation between manual and automated volume measurements of HeLa MCTS. MCTS were imaged at selected intervals of growth. Manual measurement of MCTS volume was performed by determining the average of the largest and smallest diameters using the captured images and assuming a spherical MCTS morphology. Automated volume measurement was achieved using an image recognition technique in ImageJ. Firstly, MCTS images were converted into 8-bit greyscale and the perimeter of the MCTS was recognized by an automated threshold function. The area of the 2-D MCTS mask was recorded and converted to µm^2^ by calibration using an image of known scale and subsequently used to calculate the volume.
(TIF)

Figure S2 **Validation of the acid phosphatase (APH) assay.** Results from the APH assay using HeLa (left column) and HT29 cells (right column) grown as spheroids (top row) and monolayers (bottom row) demonstrate a linear relationship between cell number and UV absorption at 405 nm. Each data point represents the mean of three independent experiments ± SD (n = 3).
(TIF)

Figure S3 **Failure of** **WST-8 assay.** Results from the WST-8 assay demonstrate a non-linear correlation between the number of cells and OD_450_ in spheroid culture.
(TIF)

Figure S4 **Fluorescence images of HT29 (a) and HeLa (b) tumor xenografts displaying markers of hypoxia (EF5 - blue) and blood vessels (CD31 - red).** Scale bars represent 100 µm.
(TIF)
