# Supplementary figures and images for: Multicellular Tumor Spheroids for Evaluation of Cytotoxicity and Tumor Growth Inhibitory Effects of Nanomedicines In Vitro: A Comparison of Docetaxel-Loaded Block Copolymer Micelles and Taxotere®
Source: PLoS One. 2013 Apr 23;8(4):e62630. doi: 10.1371/journal.pone.0062630 (PMC3633836; doi:10.1371/journal.pone.0062630)

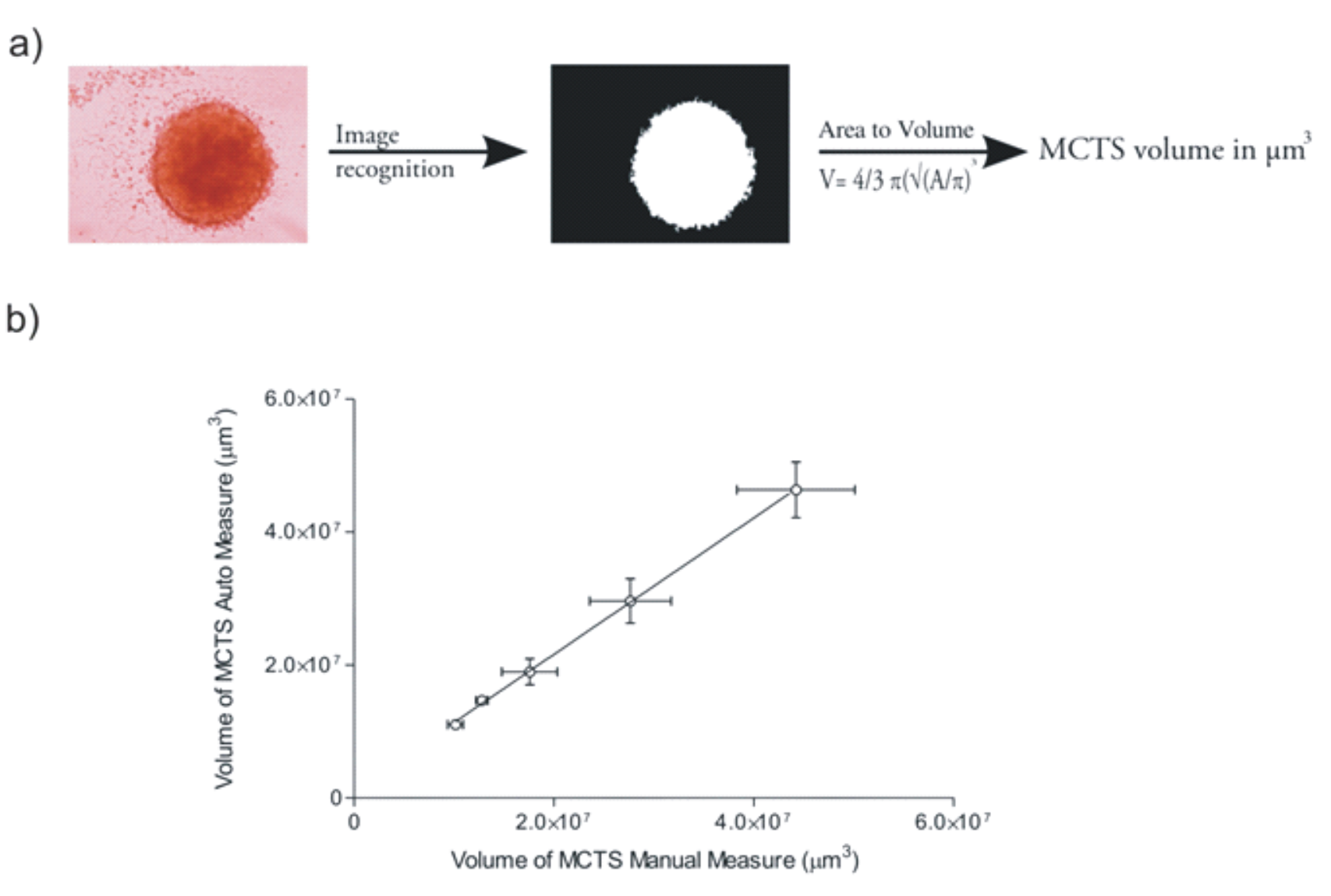

Supplement: Figure S1 — Measurement of spheroid volume. a) Schematic representation of the analysis process using a macro developed for ImageJ (version 1.44 m). b) Correlation between manual and automated volume measurements of HeLa MCTS. MCTS were imaged at selected intervals of growth. Manual measurement of MCTS volume was performed by determining the average of the largest and smallest diameters using the captured images and assuming a spherical MCTS morphology. Automated volume measurement was achieved using an image recognition technique in ImageJ. Firstly, MCTS images were converted into 8-bit greyscale and the perimeter of the MCTS was recognized by an automated threshold function. The area of the 2-D MCTS mask was recorded and converted to µm2 by calibration using an image of known scale and subsequently used to calculate the volume. (TIF) [file pone.0062630.s001.tif]

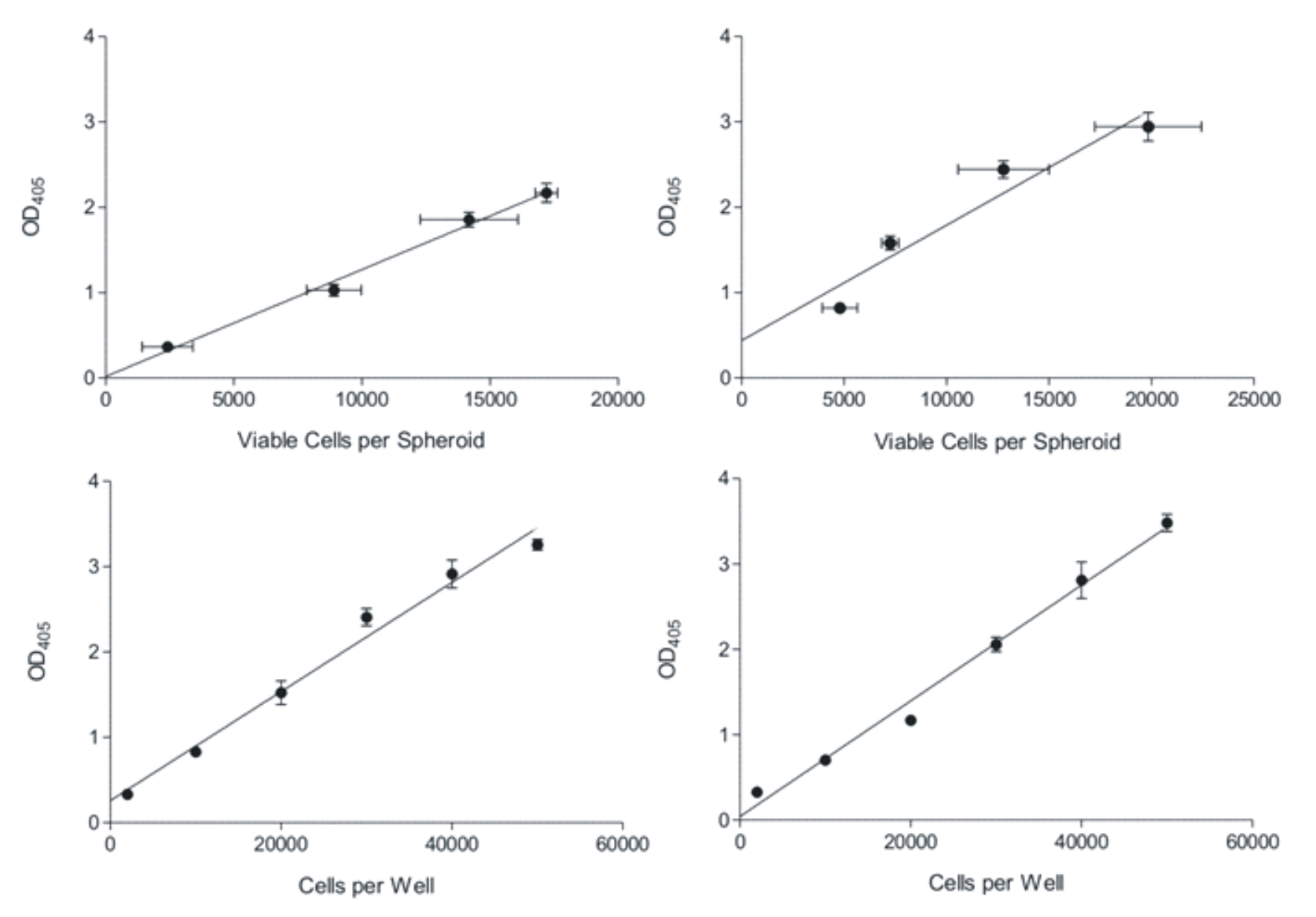

Supplement: Figure S2 — Validation of the acid phosphatase (APH) assay. Results from the APH assay using HeLa (left column) and HT29 cells (right column) grown as spheroids (top row) and monolayers (bottom row) demonstrate a linear relationship between cell number and UV absorption at 405 nm. Each data point represents the mean of three independent experiments ± SD (n = 3). (TIF) [file pone.0062630.s002.tif]

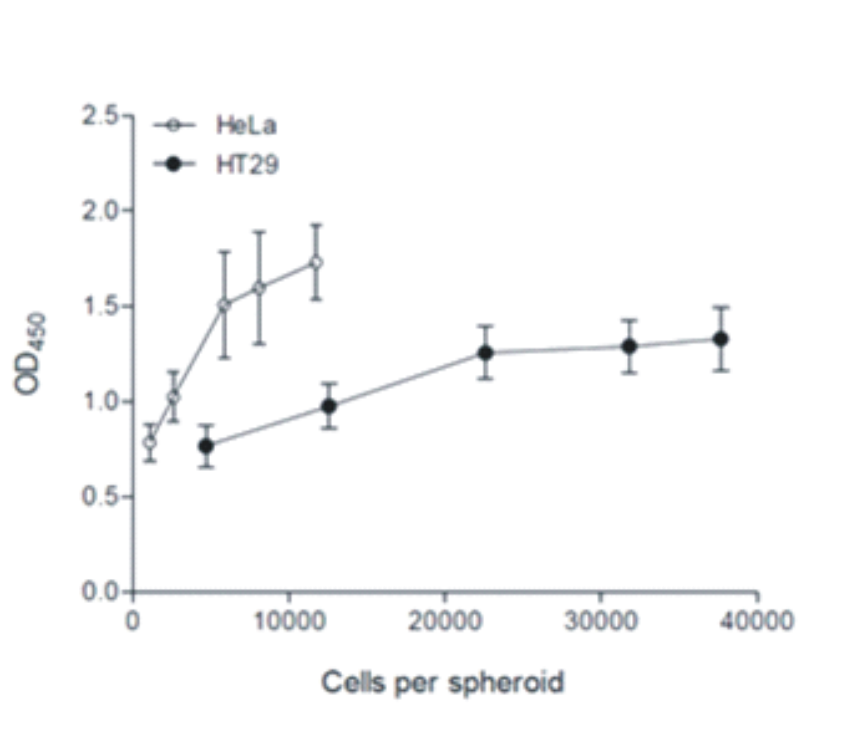

Supplement: Figure S3 — Failure of WST-8 assay. Results from the WST-8 assay demonstrate a non-linear correlation between the number of cells and OD450 in spheroid culture. (TIF) [file pone.0062630.s003.tif]

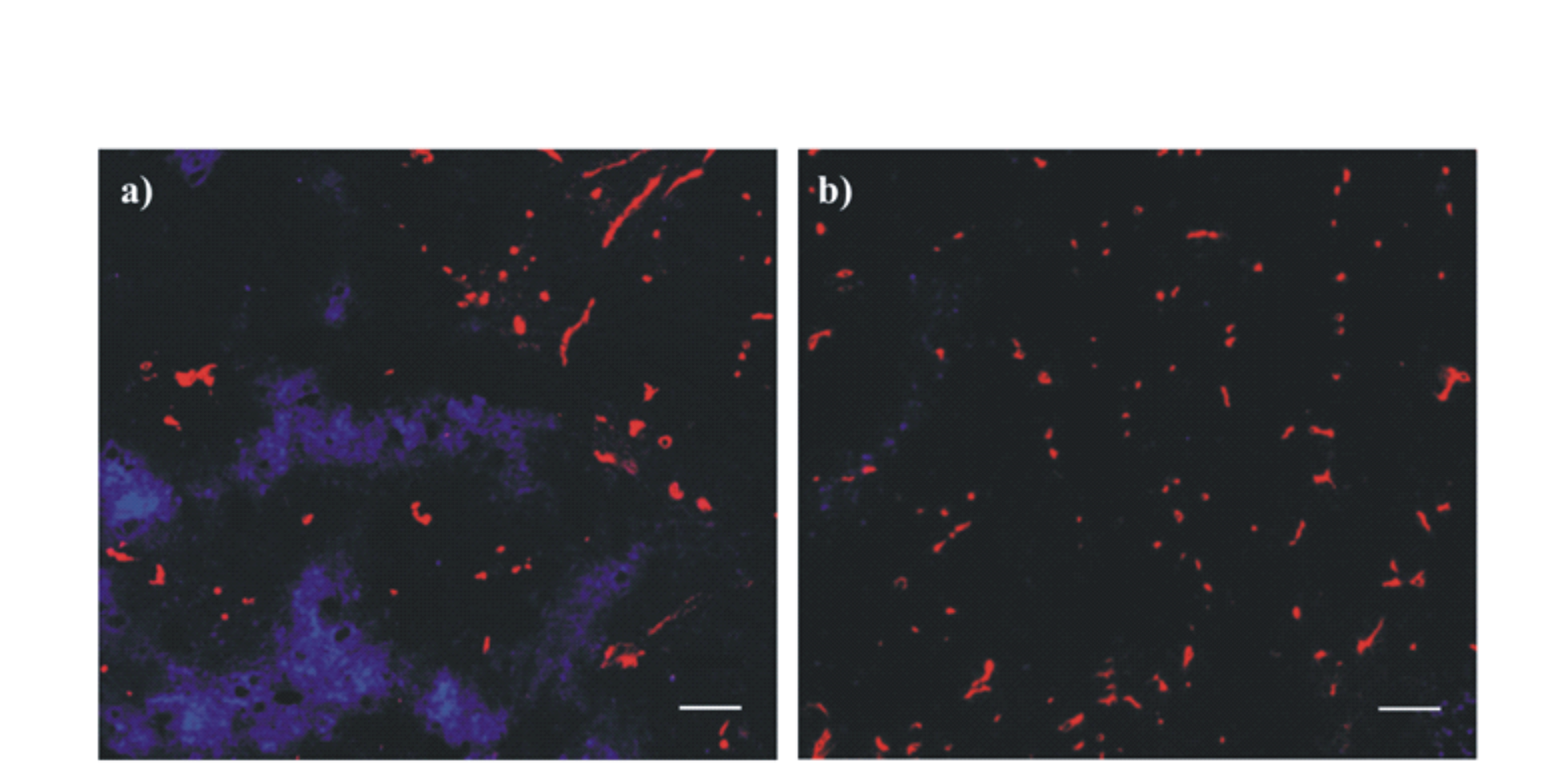

Supplement: Figure S4 — Fluorescence images of HT29 (a) and HeLa (b) tumor xenografts displaying markers of hypoxia (EF5 - blue) and blood vessels (CD31 - red). Scale bars represent 100 µm. (TIF) [file pone.0062630.s004.tif]
